# Estimating Functional Threshold Power in Endurance Running from Shorter Time Trials Using a 6-Axis Inertial Measurement Sensor

**DOI:** 10.3390/s21020582

**Published:** 2021-01-15

**Authors:** Antonio Cartón-Llorente, Felipe García-Pinillos, Jorge Royo-Borruel, Alberto Rubio-Peirotén, Diego Jaén-Carrillo, Luis E. Roche-Seruendo

**Affiliations:** 1 Health Sciences Faculty, Universidad San Jorge, 50830 Zaragoza, Spain; acarton@usj.es (A.C.-L.); royojorge@gmail.com (J.R.-B.); arubio@usj.es (A.R.-P.); leroche@usj.es (L.E.R.-S.); 2 Department of Sports and Physical Education, University of Granada, 18071 Granada, Spain; fegarpi@gmail.com; 3 Department of Physical Education, Sports and Recreation, Universidad de La Frontera, Temuco 14811, Chile

**Keywords:** aerobic, assessment, performance, physiology, technology, training, wearable

## Abstract

Wearable technology has allowed for the real-time assessment of mechanical work employed in several sporting activities. Through novel power metrics, Functional Threshold Power have shown a reliable indicator of training intensities. This study aims to determine the relationship between mean power output (MPO) values obtained during three submaximal running time trials (i.e., 10 min, 20 min, and 30 min) and the functional threshold power (FTP). Twenty-two recreationally trained male endurance runners completed four submaximal running time trials of 10, 20, 30, and 60 min, trying to cover the longest possible distance on a motorized treadmill. Absolute MPO (W), normalized MPO (W/kg) and standard deviation (SD) were calculated for each time trial with a power meter device attached to the shoelaces. All simplified FTP trials analyzed (i.e., FTP10, FTP20, and FTP30) showed a significant association with the calculated FTP (*p* < 0.001) for both MPO and normalized MPO, whereas stronger correlations were found with longer time trials. Individual correction factors (ICF% = FTP60/FTPn) of ~90% for FTP10, ~94% for FTP20, and ~96% for FTP30 were obtained. The present study procures important practical applications for coaches and athletes as it provides a more accurate estimation of FTP in endurance running through less fatiguing, reproducible tests.

## 1. Introduction

Monitoring workload is a milestone for endurance sports athletes and coaches for training prescription and competition. A wide array of physiological parameters has been targeted in search for a single biomarker truly coupled to the current intensity of the effort which, at the same time, was easy to track. These psychophysiological responses are classified as internal workload measures and mainly include the evaluation of heart rate (HR) and its derivatives (e.g., heart rate variability), blood lactate concentration, muscle oxygen saturation, and rate of perceived exertion (RPE). To date, none of them have turned out to be sensitive or handy enough to instantly quantify the athlete’s response to training stimuli [1], and multiple external (often called objective) workload metrics needed to be added to assess in-field racing intensity [2].

The development of portable global positioning system technologies (GPS) allowed the use of external metrics, such as distance and velocity, and so controlling the training pace and observing the internal responses to it became widely used as one of the best methods to assess current training stress. Unfortunately, pace is highly dependent on external conditions, such as wind, terrain, or slope, and therefore its use for quantifying intensity in the field provides results that are imprecise and is not repeatable enough. In this context, new technologies were developed in a search for an objective workload metric, giving rise to the era of mechanical power assessment.

Power output refers to the product of force and velocity, so once you are able to calculate the instant force applied to a given activity, you can accurately measure the actual workload your body is putting out. The huge step forward came when comparing power output´s instantaneous response to an increase or decrease of intensity with other traditional metrics such as heart rate, which take their time to respond. Actually, mean power output (MPO) (i.e., the averaged power output during a given time period) has proven to be more reliable and sensitive to little changes in exercise intensity than other internal and external commonly used workload indicators [3].

Accordingly, the use of power meters in cycling increased exponentially due to their capacity to assess workload considering external conditions such as wind, drafting, slope or terrain. New racing strategies (e.g., uphill pacing) emerged and power-related data also became widely used to inform decisions relating to cycling position, technique and equipment selection [4]. Through strain gauges located in the pedals, crank, or rear hub, the quantity and direction of the force applied by the cyclist, as well as the instant angular velocity, can be obtained. Therefore, power output in cycling is calculated based on the torque applied multiplied by cadence.

Analogously, running mechanical power could be quantified using force instrumented treadmills [5] which reflects forward, vertical and lateral forces applied to the integrated force plate at any given velocity. Of note, the actual external mechanical work of the foot against the ground is negligible, so the term power output in running represents an abstraction of the mechanical power theoretically applied to the runner´s centre of mass. Despite its accuracy, force instrumented treadmills are not usable for an in-field evaluation, thus, some commercial companies started to develop wearable power meters for running. These novel devices can estimate the force applied by the subjects derived from their height, body mass, and velocity, using GPS technology in outdoor environments and IMUs when indoors [6]. In fact, a model proposed recently by Jenny and Jenny [7] supports that the mechanical energy for steady flat running could be expressed as the sum of the energy employed to counteract aerodynamic drag and the energy dissipated to produce vertical oscillation and braking.

In the aforementioned mathematical approach [7] the rate of mechanical energy (i.e., the power output) dissipated to break through the air can be estimated knowing both the runner and the wind´s velocity, and the runner and the air´s density. Energy dissipation due to braking ground reaction forces may be estimated assuming the sine wave movement described by the runner’s centre of mass, following the spring-mass model presented by Blickhan [8]. Finally, dissipation in vertical oscillation is calculated based on spatiotemporal parameters (speed, step rate, ground contact time) and a potential energy recovery factor. This factor depends on the athlete´s ability to reuse the elastic energy stored during the braking phase, into kinetic energy during the propulsion phase. As this condition is highly variable between individuals, it represents the main concern within the entire model.

Given the complexity of testing the validity of wearable running power meters against a gold standard method (i.e., instrumented treadmill), a recent study from Cerezuela-Espejo [9] compared PO obtained with five commercially available portable devices and two of the mathematical models applied to theoretically calculate running power. The results showed the closest agreement corresponded to the Stryd^TM^ system among all investigated devices.

Regarding the agreement between mechanical and metabolic power, another published comparison between five portable devices [10] showed a promising correlation (r^3^ = 0.911, SEE = 7.3%) between power output data obtained with the Stryd^TM^ foot pod and oxygen consumption as a measure of energy expenditure, both in laboratory (i.e., treadmill running) and the in-field conditions, even when changes in body mass and slope were applied [10]. Table 1 summarizes the scientific evidence found on the use of the main commercially available power meters, and further information is available in a recently published scoping review on sensors for running power output assessment [11].

Regarding the application of power output data to determine training stress and intensity zones, Allen and Cogan [16] proposed a performance index known as the Functional Threshold Power (FTP). It refers to the highest MPO maintained in a quasi-steady state for 60 min (FTP_60_) without the onset of fatigue [16]. FTP has demonstrated its validity as a surrogate for lactate threshold (LT) [17] and maximum lactate steady state (MLSS) [18,19]. LT is defined as the maximum intensity preceding an exponential rise in blood lactate values during an incremental test [20], being addressed that during a continuous effort at LT blood lactate concentration steadily rises [17]. However, MLSS refers to the maximum workload that can be maintained over time without continual blood lactate accumulation (i.e., 45–70 min) [18,21]. Additionally, MLSS demonstrated to better predict endurance performance than maximum oxygen uptake (VO2max) in trained athletes [20]. Thus, FTP is considered a good indicator of the main physiological events of the aerobic-anaerobic transition for endurance activities and therefore it has been commonly used lately to determine training intensities (i.e., training zones) and quantify athletes’ responses to training stimuli.

Although FTP_60_ is a highly reproductible and widely accepted method to assess aerobic condition [11], less time-consuming time trials (TT) are demanded for in-season regular evaluations. The 20-min TT (FTP_20_) has become the most popular simplified test to predict FTP_60_ [22,23]. Allen and Cogan [16] set 95% of the MPO obtained in FTP20 as a predictive value for FTP_60_ in cycling. Thereafter, a few studies [23,24,25] confirmed this 95% individual correction factor (ICF% = FTP_60_/FTP_20_) between both TTs, whereas some others [26,27,28] found stronger associations between FTP_20_ and MLSS subtracting ~10% to the MPO achieved during the TT, instead of 5%. Furthermore, other TTs ranging from 3 to 30 min were proposed as MLSS predictors of FTP_60_ [24,29]. Despite an overall moderate to high level of agreement between these simplified TTs and FTP_60_, most referred to cycling.

The advent of wearable running power meters allows the transfer of knowledge (and the FTP60 assessment) from cycling to running. Unlike other parameters such as HR, VO2max or RPE, the physiological response of blood lactate showed no differences between cycling and running at constant submaximal velocities [30]. Therefore, the determination of FTP_60_ as a valid substitute of MLSS would be a key point for endurance running. Unfortunately, knowledge about the level of agreement and correction factor between simplified TTs and FTP_60_ in running is unknown. Nevertheless, the development of novel technologies, such as running power meters, may help evaluate athletes’ functional performance and monitor changes over time. A recent study confirmed a linear power–velocity relationship in running for maximal and submaximal protocols [13]. This enables the prediction of MPO at different submaximal running velocities using the two-point method, underlining the need to accurately determine the relationship between simplified FTP tests and FTP_60_ method.

Up to date, there are no studies which investigate the validity of simplified running test to predict FTP_60_. Consequently, this study aims to analyse the level of agreement between mean power values during three different running TTs (10-, 20- and 30-min) compared to a 60-min TT, and to establish the correction factor for each simplified FTP running test. Considering the high concordance reported in cycling, we hypothesized that the 10-, 20-, and 30-min TTs (i.e., FTP_10_, FTP_20_ and FTP_30_) would have a good level of agreement with FTP_60_ in running, and they could be valid substitutes of the FTP_60_.

## 2. Materials and Methods

### 2.1. Participants

Twenty-two recreationally trained male endurance runners (age: 34.0 ± 7.5 years; height: 1.76 ± 0.04 m; body mass: 71.1 ± 5.8 kg; BMI: 22.9 ± 1.5 kg/m^2^) voluntarily participated in this study. All participants met the inclusion criteria: older than 18 years old, able to run 10 km under 40 min, used to running treadmill, and free from injuries the last 6 months before data collection. After receiving detailed information of the study, participants signed an informed consent form, complied with the ethical standards of the World Medical Association’s Declaration of Helsinki (2013), prior to participation. It was made clear that participants were free to leave the study at any point. The study was approved by the local Ethics Committee.

### 2.2. Procedures

The study protocol was executed between March and June 2019 in the laboratory of biomechanics of the founding institution. Participants were asked to complete four submaximal time trials (10, 20, 30, and 60 min) attempting to cover the longest distance they could on a motorized treadmill (HP cosmos Pulsar 4P; HP cosmos Sports & Medical, Gmbh, Nußdorf, Germany). During all tests slope was maintained at 0° and ventilation was assured using two industrial fans located laterally at 2 m distance from both sides of the treadmill. Fluid intake was ad libitum while temperature and humidity were controlled with a wireless weather station (Ea2 LABS DE903) and kept between 18 and 20 °C and 50–60%, respectively.

Participants were encouraged to maintain their normal dietary pattern and to avoid ergogenic aids and severe physical activity for 48 h before the tests, which were scheduled at the same time of the day and performed within a 1-week separation interval. Trial order was randomly set, and participants wore their usual running shoes during the entire protocol to reproduce their usual performance.

### 2.3. Materials and Testing

Body height (cm) and mass (kg) were measured at the beginning of the first testing session using a precision stadiometer and weighing scale (SECA 222 and 634, respectively, SECA Corp., Hamburg, Germany). Additionally, personal best time in a 10-km race within the last 6 months were recorded and all the athletes were instructed on the use of the RPE scale [31].

Before each time trial, participants’ body mass was re-evaluated to adjust the power data collected. A standardized 8-min protocol (4-min at self-selected velocity and 4-min approaching their expected velocity for the trial) was completed for avoiding the accommodation effect of treadmill running [32].

During the tests, participants received verbal encouragement from the same researcher to complete the longest distance they can, and slight velocity variations were allowed along the entire protocol. HR was continuously monitored using a chest belt (Polar, FS2c, Kempele, Finland), and RPE was assessed every 5 min until the end of the test. MPO (in W) was calculated using the Stryd™ power meter (Stryd Power meter, Stryd Inc. Boulder, CO, USA) attached to the shoelaces.

After each test, maximum HR and total distance covered was recorded and mean velocity calculated. Data from Stryd™ power meter were obtained from their website (https://www.stryd.com/powercenter/analysis) into .fit file. Then, data were analyzed using a free-license software (Golden Cheetah, version 3.4) and exported as .csl file into Excel^®^ (2016, Microsoft, Inc., Redmond, WA, USA). Absolute MPO (W), normalized MPO (W/kg), and standard deviation (SD) were calculated for each time trial.

### 2.4. Stryd^TM^ System

This device is a lightweight (9.1 g) carbon fibre-reinforced foot pod that includes a 6-axis inertial motion sensor (3-axis gyroscope, 3-axis accelerometer). With a sampling rate of 1000 Hz, the 6-degrees-of-freedom device senses forward, vertical and lateral accelerations and angular velocities of rolling, pitching and yawing, to infer ground reaction forces and orientation. Integrating accelerations, the sensor gets velocities, and doble integrating it gets positions. Assuming the lateral motion as negligible in running, inverse dynamics might be applied to model vertical and horizontal forces from the positional and velocity changes of the device in each step.

As it has been roughly explained by the Stryd^TM^ team in a recent white paper on their web site (https://blog.stryd.com/tag/validation-white-papers/), accounting vertical decelerations and accelerations, the height and body mass of the runner, and the flight time between steps, an algorithm estimates the vertical displacement of the runner´s center of mass, and thus, its potential energy variation is available to calculate vertical power (named as form power by the Stryd^TM^ manufacturers). While moving forward, a runner losses momentum on foot impact and gains it during take-off. The change in kinetic energy between events is given by the difference between the minimum and maximum instant velocities and the body mass of the runner, allowing forward power calculation. Finally, external PO is the time derivative of the summation of changes in potential and kinetic energy.

As a result, this wearable power meter additionally provides real time measures of vertical oscillation, elevation, distance and ground contact time, and interesting power-related metrics (i.e., averaged elapsed power, maximal power, form power, leg spring stiffness and running effectiveness). Of note, the presented model does not account for the internal power the runner needs to relocate the limbs in relation to the center of mass. However, the manufacturers presume the newest version of the product include a sensor to detect wind force and direction that might allow a better estimation of these internal forces.

Although the actual strategy Stryd^TM^ use to isolate the sensor to avoid measurement noise, and the algorithmic computation to process raw data still undisclosed by the company as part of their knowhow, this system has demonstrated reliable to assess running spatiotemporal parameters in indoor setting compared to 3D motion analysis [33] and the OptoGait infrared system [13]. Furthermore, this sensor has shown moderate to excellent intra-system reliability for all measures through trail running bouts [34].

Presumably, the algorithm employed by the company to calculate power output might be based on the model proposed by Jenny and Jenny [7] and assumes certain controversial simplifications related to the athletes´ individual energy recovery factor. However, a recent study [10] evaluated the agreement between energy expenditure (i.e., oxygen consumption) and Stryd´s PO data under different conditions (i.e., athletic track, flat, and inclined treadmill running) showing strong correlations between variables in all conditions (r ≥ 0.911, SEE ≤ 7.3%).

### 2.5. Statistical Analysis

Descriptive statistics are represented as mean (±SD). Before analysis, normal distribution and homogeneity were confirmed through the Shapiro-Wilk and Levene’s test, respectively. A repeated measures analysis of variance (ANOVA), with post-hoc Bonferroni test, was conducted to compare the acute response (i.e., running speed, MPO and RPE) to the different time trials conditions (i.e., 10, 20, 30 and 60 min). Additionally, the level of agreement between MPO reported during shorter time trials (i.e., 10-min, 20-min and 30-min) and the reference trial (i.e., 60-min) was examined. Therefore, a Pearson correlation analysis was performed and intra-class correlation coefficients (ICC) with 95% confidence interval (CI) were calculated (i.e., 10-min, 20-min and 30-min vs. 60-min). The following criteria were adopted to interpret the correlations magnitude between variables: <0.1 (trivial), 0.1–0.3 (small), 0.3–0.5 (moderate), 0.5–0.7 (large), 0.7–0.9 (very large) and 0.9–1.0 (almost perfect) [35]. Based on the characteristics of this experimental design and following the guidelines reported by Koo and Li [36], the authors decided to conduct a “two-way random-effects” model (ICC [2,k]), “mean of measurements” type, and “absolute” definition for the ICC measurement. The interpretation of the ICC was based on the benchmarks reported by a previous study [37]: ICC < 0 (poor), 0–0.20 (slight), 0.21–0.40 (fair), 0.41–0.60 (moderate), 0.61–0.80 (substantial), and >0.81 (almost perfect). Finally, a linear regression analysis was conducted between 60-min MPO and MPO during shorter trials. The level of significance used was *p* < 0.05. Data analysis was performed using SPSS (version 23, SPSS Inc., Chicago, IL, USA).

## 3. Results

**Figure 1:**
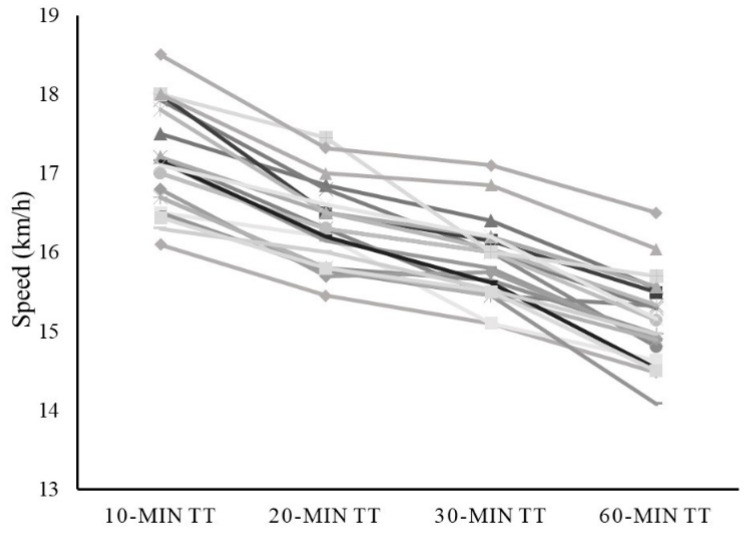
Individual average running speed for each time trial (i.e., 10 min, 20 min, 30 min and 60 min). Each athlete’s speed mean values are represented in a different color line.

**Figure 2:**
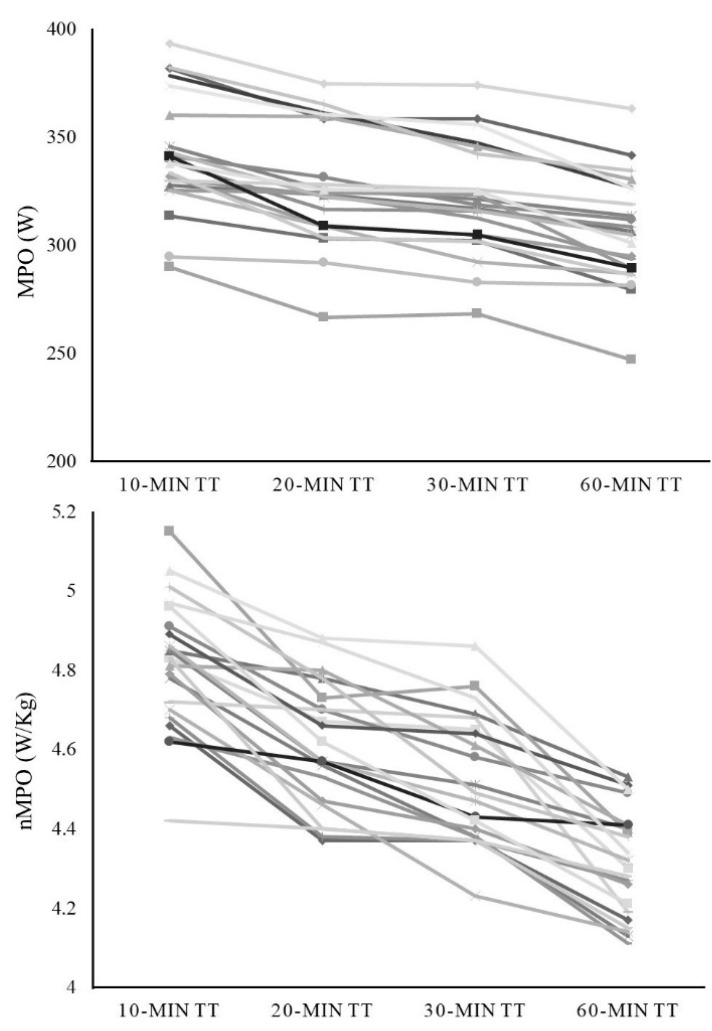
Individual analysis of the power output during running, in absolute (upper panel) and relative values (lower panel) (i.e., 10-min, 20-min, 30-min and 60-min time trials). MPO: mean power output; nMPO: normalized mean power output.

Table 2 shows the acute response of the examined variables to the different running protocols. The repeated measures ANOVA reported significant differences between tests in running speed (*p* < 0.001), MPO in absolute and relative values (*p* < 0.001) and RPE (*p* = 0.011). After post-hoc testing, differences between each test were found in all variables, apart from RPE, with the 30-min trial showing lower values than the rest of trials. The individual average running speed and MPO for each time trial are shown in Figure 1 and Figure 2, respectively.

The level of agreement of power values obtained during running protocols with different durations, as compared to 60-min time trial, was examined (Table 2). For mean power values (W), all the durations (i.e., 10, 20, and 30 min) showed an almost perfect correlation (r > 0.9), whereas the ICC was moderate with data obtained from the 10-min trial (ICC = 0.647), and very large with 20-min and 30-min trials (ICCs = 0.839 and 0.899, respectively). Regarding the normalized mean power values (W/kg), the correlation with data reported during the 60-min trial was very large (r = 0.720 for 10-min trial, 0.868 for 20-min trial and 0.859 for 30-min trial) and the ICCs revealed slight (ICC = 0.188), moderate (ICC = 0.432) and substantial (ICC = 0.625) coefficients for 10-min, 20-min and 30-min time trials, respectively, compared to 60-min protocol. Additionally, the ICF% and CI for each TT were calculated for both MPO and normalized MPO (Table 3).

**Figure 3:**
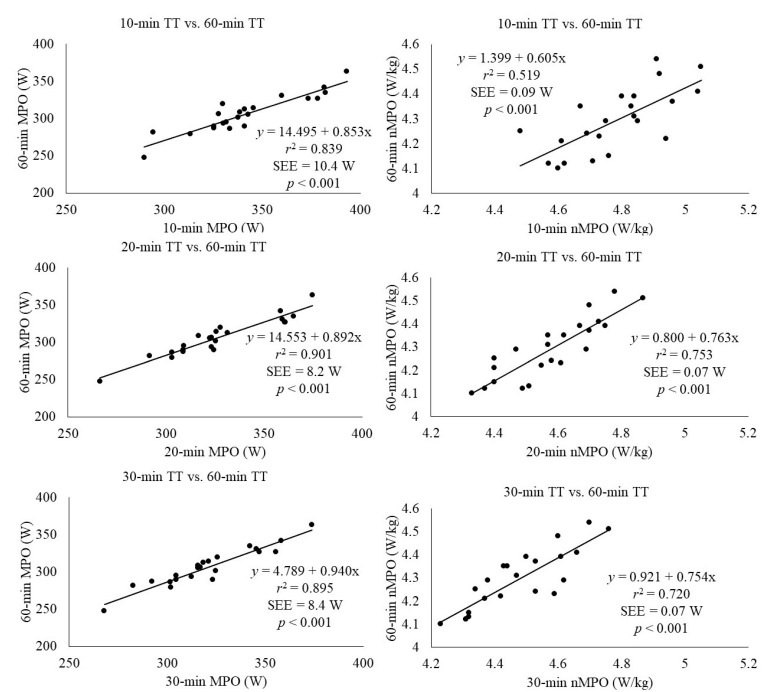
Association between the mean power output achieved during a 60-min time trial (TT) and the MPO achieved during 10-min, 20-min and 30-min TTs in trained endurance runners. (Absolute values in left column and relative values in right column). The average value of each runner was used for each TT. Circles indicate individual data. The solid lines represent the predictive linear regression model between 60-min TT and the shorter TTs. SEE: standard error of estimate; MPO: mean power output; nMPO: normalized mean power output.

The regression analysis revealed a significant association (*p* < 0.001) between 60-min MPO and the MPO reported during shorter trials (i.e., 10-, 20- and 30-min) (Figure 3). The 10-min MPO obtained the lowest r^2^ (r^2^ = 0.839) and the greater SEE (10.4 W), whereas almost identical values were obtained in 20- and 30-min (r^2^ = 0.901, SEE = 8.2 W; r^2^ = 0.895, SEE = 8.4 W, respectively). Regarding normalized MPO (Figure 1), a significant association (*p* < 0001) was found between 60-min values and those reported during shorter trials (i.e., 10-, 20- and 30-min), with stronger associations found with longer time trials (r^2^ = 0.519, SEE = 0.09 W for 10-min; r^2^ = 0.753, SEE = 0.07 W for 20-min and; r^2^ = 0.720, SEE = 0.07 W for 30-min).

## 4. Discussion

This study sought to analyze the level of agreement between the 60-min TT and three shorter TTs. The main finding of this study is that all simplified FTP trials analyzed (i.e., FTP_10_, FTP_20_ and FTP_30_) showed a significant association with the FTP_60_ for both MPO and normalized MPO, exhibiting stronger correlations with longer TTs (i.e., FTP_20_ and FTP_30_). Moreover, the ICF% determined for each TT were 89.6%, 93.6%, and 95.5% of the MPO from the FTP_10_, FTP_20_ and FTP_30_, respectively; and 89.8%, 93.7%, 95.6% from the aforementioned TTs when MPO was normalized to the daily athletes’ body mass.

### 4.1. FTP_60_

To the best of the authors’ knowledge, this is the first study aimed to establish the relationships between FTP_60_ and shorter TTs based on running power metrics. An average MPO of 306.15 ± 25.33 W was obtained for the FTP60 at a mean speed of 15.12 ± 0.56 km/h in trained endurance runners. This result seems to be in line with a previous study [21] which addressed a MPO of 285.2 ± 25.6 W in a group of trained athletes during a 3-min run at 15 km/h. Considering normalized MPO, our results for the FTP_60_ test showed a mean of 4.29 ± 0.13 W/kg. In a previous study [28], the average normalized MPO reported at their LT pace was 4.4 ± 0.5 W/kg. As the MPO in this study was assessed at their calculated LT pace, comparison between both studies should be cautiously considered as the MPO at MLSS might be considerably lower. Additionally, a normalized MPO of 4.29 W/kg is considered as excellent in cycling [16] and therefore our results are supported by previous studies for this level of endurance athletes [16].

### 4.2. FTP_10_

The agreement between FTP60 and FTP10 ranged from very large to almost perfect for relative and absolute power output assessment, respectively. In addition, the ICF% obtained was ~90%, for both MPO and normalized MPO. The average MPO obtained was 341.73 ± 27.19 W, the normalized MPO was 4.78 ± 0.15 W/kg, and the mean velocity of the test was 17.16 ± 0.56 km/h. These results slightly exceed those found in previous studies [13,15], although the speeds are not entirely comparable. Despite the results for FTP10 exhibiting the lowest association with FPT60 within the three TTs tested, previous studies confirmed a good association between short TTs (i.e., ≤10 min) and LT derivatives [22,38,39]. Despite the good association found between FTP_10_ and FTP_60_, our results were slightly weaker compared with longer TTs (i.e., FTP_20_ and FTP_30_). Of note, these studies [38,39] also found broader differences between calculated MLSS and FTP estimated with shorter TTs (i.e., ≤10-min) than with longer TTs (i.e., ≥20-min). These discrepancies might partially be explained as a result of differences in the athletes’ metabolic profile, as the MPO of shorter TTs is more likely to be achieved with a higher participation of the anaerobic metabolism [40]. Despite these controversial precedents, we found a large association between FTP_10_ and FTP_60_, and therefore it could be useful for running practitioners aiming to execute a rapid test that fairly identifies training zones and adaptations.

### 4.3. FTP_20_

Regarding FTP_20_ association with FTP_60_, our results showed a very large to almost perfect correlation for both absolute and normalized MPO. The ICF% found was 93.6%, which contradicted the 95% established by Allen and Coggan [16] and supported others [23,24,25]. Contrary, recent studies [26,27,28] pointed that the well-accepted rule of subtracting 5% from the FTP_20_ MPO is not a “one-size-fits-all” accurate method for FTP_60_ estimation as it may differ depending on the athlete’s level of performance. Our findings support this statement as an overestimating trend would affect our non-elite athletes when 95% of the FTP_20_ is applied. Furthermore, Valenzuela et al. [24] tested two different cyclist groups (i.e., trained and recreational) and claimed that lower fitness status could result in FTP_60_ overestimation as only the trained group matched the 95% adjustment for FTP20. Moreover, MacInnis described an ICF% of 90% for FTP_20_ in 8 well-trained cyclists [39], whereas Lillo-Bevia tested 11 trained cyclist and triathletes finding an ICF% of 91% [27]. It should be considered that the aforementioned studies did not match the 95% adjustment [26,27,28] followed by a modified warm-up protocol (i.e., ≤15 min at self-selected pace), whereas those that reported a 95% correction between test [23,24,25] strictly followed the warm-up protocol originally proposed by Allen and Coggan [16] (50 min, including three 1-min accelerations and a 5-min all-out effort). Therefore, it was hypothesized that the type of warm-up selected may explain the differences between studies [25]. As a 50-min warm-up protocol would jeopardize running performance, and the final purpose of a simplified TT is to reduce the duration and fatigue induced by the testing protocol itself, a modified warm-up protocol was adopted [28] seeking for a more practical approach.

The average MPO obtained in our FTP_20_ (326.9 ± 26.97 W) and the normalized MPO (4.58 ± 0.15 W/kg) are similar to those found in previous studies [13,15]. Of note, the recording time in these studies lasted two and three min, respectively. Thus, the minor mean differences found might be attributed to the fatigue induced along the 20-min test.

### 4.4. FTP_30_

A strong association was found between FTP_30_ and FTP_60_ with substantial to very large confidence interval for relative and absolute values. Our results also identify an ICF% of ~96%, an MPO of 320.63 ± 25.51 W and a normalized MPO of 4.47 ± 0.15 W/kg. Previous studies [13,15] evaluated absolute MPO and normalized MPO at similar velocities (~16 km/h) showing similar results for same-level trained runners. Unfortunately, to our knowledge there are no previous studies assessing the relationship between both tests. It is worth mentioning that many studies conducted several constant-velocity 30-min TTs as a valid method to determine MLSS [27], nevertheless, it is hard to establish a comparison because in these TTs the participants were not encouraged to cover the longest distance they could but to keep a previously fixed PO. Despite this, our results showed to be consistent and allow an accurate prediction of FTP_60_. However, as the results found little differences between FTP_20_ and FTP_30_ for their correlation with FTP_60_ (ICCs = 0.839 and 0.899, respectively), it would be advisable to opt for the shortest one to reduce both time needed and stress caused in the athletes.

Although the use of IMU technology for estimating running power is quite recent, its rapid development might be a promising step forward in the field of exercise physiology. Added to heart rate monitors and GPS technology, real-time power output data could help for a better understanding of the cardio-respiratory and skeletal muscle responses to different intensity runs. In order to effectively monitor the physical impact of running through portable running power meters, an accurate determination of the main physiological boundaries is mandatory. Whereas previous works validated the use of power meters in cycling for FTP assessment [22,41], analogous evidence for running devices is lacking. Despite up to date no studies have investigated the concurrent validity of running power meters, Olaya et al. [42] found and almost perfect association between PO (measured with Stryd^TM^ system) and pace data (measured via GPS technology) in their comparison of five methods to determine the FTP during level running. Additionally, in a recent review on sensors for running power [11] it has been stated that the Stryd foot pod has the highest repeatability and agreement with metabolic power among all commercially available portable running power devices. In this context, a broader framework related with the validity and applicability of Stryd^TM^ system is becoming of relevance.

Despite the findings reported here, there are some limitations to consider. First, blood lactate concentration during the tests was not directly assessed. Additionally, the MPO corresponding to the MLSS (i.e., the FTP) was not validated through other calculation methods, assuming that the time-to-exhaustion at their MLSS intensity should be approximately 60 min for a homogenous sample of trained athletes [19]. However, constant-duration time trials were conducted as they have proven to be more reliable than time-to-exhaustion protocols [43]. Additionally, we assessed RPE and HR during all tests in order to control performance intensity. Despite lower mean values for RPE during the 30-min trial, the physiological and perceptual responses to simplified TTs confirmed an intensity above MLSS, whereas most of the FTP_60_ was performed at an intensity equal to or slightly above MLSS. Maximal RPE of every test was ≥18, and HR peak differ ≤5% between tests. Regarding data generalization, only male trained runners participated in the study, preventing the possible sex differences analysis. The sample size was selected by convenience. Nevertheless, a post hoc analysis of the achieved power for this sample was conducted (G*Power software vs. 3.1) revealing a moderate to high power (~0.6). Ultimately, although all participants were familiar with its use, the entire experimental protocol was conducted on a motorised treadmill. Thus, the accuracy of the ICF% could be reduced when applied in field-based conditions. Notwithstanding these limitations, the current study highlights that the 10-, 20-, and 30-min TTs are valid for the estimation of FTP_60_ in trained endurance runners fitting FTP_20_ and FTP_30_ better for this purpose than FTP_10_.

## 5. Conclusions

The results obtained here showed that the three simplified TTs (i.e., FTP_10_, FTP_20_ and FTP_30_) can provide good estimations of MPO and normalized MPO achieved during a 60-min submaximal TT (i.e., FTP_60_). Moreover, as FTP_10_ showed a lower correlation, and FTP_20_ and FTP_30_ exhibited similar results, the FTP_20_ would be the preferred simplified TT to assess FTP_60_ in endurance runners, as it is less prone to fatigue. Additionally, an ICF% of ~94% for the FTP_20_ was found to be more compliant with FTP_60_ in recreationally trained runners than the well-accepted 95%.

FTP is an essential parameter in prominent commercially available software such as TrainingPeaks for both determining training intensity (i.e., intensity factor) and monitoring training load (i.e., training stress score). Moreover, the ICF% revealed for each test (~90% for FTP_10_, ~94% for FTP_20_ and ~96% for FTP_30_) may lead practitioners to an accurate evaluation of FTP through less fatiguing, more easily reproducible tests. However, the predictive value of the simplified TTs reported here might differ between laboratory and on-field conditions.

Future research should focus on the on-field repeatability of the algorithms reported hereabouts in order to incorporate them to the endurance runners´ performance assessment. Once validated it might lead coaches and athletes’ decisions for training and racing, as it happens before with cycling. Additionally, shorter TT may be also included for a better understanding of the individual aerobic–anaerobic profile of the athletes. Finally, the response of female athletes as well as different levels of performance runners should be evaluated, for a better adjustment of the aforementioned algorithms.

## Figures and Tables

**Table 1:** Studies (*n* = 5) evaluating the use of wearable power meters or using their power output data during running protocols.

	System Used	Device & Location	Aim	Results
Cerezuela-Espejo et al. (2020) [10]	RunScribe	Attached to shoelaces and paired to a Garmin Forerunner 235	To compare 4 power meter devices in terms of repeatability and concurrent validity between P data and oxygen consumption (VO_2_).	Fair repeatability indoor: SEM ≥ 30.1 W, CV ≥ 7.4%, ICC ≤ 0.709, and SEM ≥ 59.3 W, CV ≥ 14.8%, ICC ≤ 0.563. Low correlation between P and VO_2_ (r ≥ 0.582, SEE ≤ 13.7%)
	Garmin Running Power	Garmin TRI^TM^ heart rate (chest) monitor band and Garmin Forerunner 935 watch Kansas, USA		Low repeatability indoor: SEM ≥ 47.0 W, CV ≥ 9.4%, ICC ≤ 0.495, fair repeatability outdoor: SEM ≥ 24.5 W, CV ≥ 7.7%, ICC = 0.823. Low correlation between P and VO_2_ (r ≥ 0.539, SEE ≤ 17.5%)
	Polar Vantage V	Sport watch on the wrist. GPS and barometer sensors		Low repeatability outdoor: SEM ≥ 40.6 W, CV ≥ 14.5%, ICC = 0.487. Good correlation between P and VO_2_ (r = 0.841, SEE = 9.7%)
	Stryd (foot pod)	Attached to shoelaces and paired to a Garmin Forerunner 235 or a mobile phone		Best repeatability values both indoor: SEM ≤ 7.4 W, CV ≤ 2.8%, ICC ≥ 0.980, and outdoor: SEM ≤ 12.5 W, CV ≤ 4.3%, ICC ≥ 0.989. High correlation between P and VO_2_ (r^3^ ≥ 0.911, SEE ≤ 7.3%)
García-Pinillos et al. (2019) [12]	Stryd (foot pod)	Attached to shoelaces and paired to a mobile phone	To evaluate the stability of power output data while running at a constant comfortable velocity on a motorized treadmill.	P running at an easy pace is a stable metric with negligible differences, between intervals ranging from 10 to 180 s.
García-Pinillos et al. (2019) [13]	Stryd (foot pod)	Attached to shoelaces and paired to a mobile phone	To confirm the linear P-V relationship in endurance runners at submaximal velocities, and to predict P values with the “two-point method”.	Two distant velocities were able to provide P with the same accuracy than the multiple-point method.
Austin et al. (2018) [14]	Stryd (foot pod)	Attached to shoelaces and paired to a Garmin Fenix 3 watch	To determine the correlations between P and running economy at LT pace.	RE is positively correlated with Stryd’s power output data, however it may not be precise enough to notice changes in running economy
Aubry et al. (2018) [15]	Stryd (chest strap)	Stryd Pioneer 3-axial accelerometer chest band in conjunction with a mobile phone (with GPS).	To assess if running power could be a valid surrogate of metabolic demand (VO_2_) in a population of different level of training runners.	Running power is not a valid surrogate of the energy cost of running in a mixed ability population of runners.

LT: blood lactate thresholds; VO2: oxygen uptake; P: power; V: velocity; SEM: standard error of measurement; CV: coefficient of variation; ICC: intraclass correlation coefficient; RE: running economy.

**Table 2:** Acute response (mean, SD) to the different running time trials.

	10-min Trial	20-min Trial	30-min Trial	60-min Trial	Main Effect of Test *p*-Value
Running speed (km/h^−1^)	17.16 (0.65) ^b,c,d^	16.33 (0.53) ^a,c,d^	15.88 (0.50) ^a,b,d^	15.12 (0.56) ^a,b,c^	<0.001
Mean power output (W)	341.73 (27.19) ^b,c,d^	326.90 (26.97) ^a,c,d^	320.63 (25.51) ^a,b,d^	306.15 (25.33) ^a,b,c^	<0.001
Normalized mean power output (W/kg^−1^)	4.78 (0.15) ^b,c,d^	4.58 (0.15) ^a,c,d^	4.47 (0.15) ^a,b,d^	4.29 (0.13) ^a,b,c^	<0.001
RPE (6–20)	19.27 (0.83) ^c^	18.95 (0.84)	18.64 (0.73) ^a,d^	19.27 (0.88) ^c^	0.011

^a^ indicates significant differences regarding 10-min trial after post-hoc testing; ^b^ indicates significant differences regarding 20-min trial after post-hoc testing; ^c^ indicates significant differences regarding 30-min trial after post-hoc testing; ^d^ indicates significant differences regarding 60-min trial after post-hoc testing.

**Table 3:** Level of agreement between power output obtained during different running based-time trials regarding the reference duration (60-min time trial).

		Mean Power (W)	Normalized Mean Power (W/kg)
10-min vs. 60-min	Correlation (r-coefficient)	0.916 ***	0.720 ***
	ICC (95% CI)	0.647 (−0.084–0.909)	0.188 (−0.046–0.566)
	ICF% (CI)	89.6 (88.2–90.9)	89.8 (88.8–90.7)
20-min vs. 60-min	Correlation (r-coefficient)	0.949 ***	0.868 ***
	ICC (95% CI)	0.839 (−0.120–0.964)	0.432 (−0.061–0.808)
	ICF% (CI)	93.6 (92.5–94.7)	93.7 (93.1–94.4)
30-min vs. 60-min	Correlation (r-coefficient)	0.946 ***	0.859 ***
	ICC (95% CI)	0.899 (−0.089–0.976)	0.625 (−0.152–0.895)
	ICF% (CI)	95.5 (94.3–96.6)	95.9 (95.2−96.6)

*** *p* < 0.001; ICC: intra class correlation coefficient; ICF%: individual correction factor (%); CI: confidence interval.

## Data Availability

The data presented in this study are available on request from the corresponding author. The data are not publicly available due to authors preferences.
